# Life on Mars? The physiological perspective

**DOI:** 10.1113/EP093492

**Published:** 2026-02-04

**Authors:** Ronan M. G. Berg, Damian M. Bailey

**Affiliations:** ^1^ Centre for Physical Activity Research Copenhagen University Hospital—Rigshospitalet Copenhagen Denmark; ^2^ Department of Clinical Physiology and Nuclear Medicine Copenhagen University Hospital—Rigshospitalet Copenhagen Denmark; ^3^ Department of Clinical Medicine, Faculty of Health and Medical Sciences University of Copenhagen Copenhagen Denmark; ^4^ Neurovascular Research Laboratory, Faculty of Life Sciences and Education University of South Wales Pontypridd UK; ^5^ Bexorg, Inc. New Haven Connecticut USA

Few scientific discoveries capture the imagination quite like possible signs of life beyond Earth. For physiologists, these findings are more than astronomical curiosities: they challenge us to consider how the fundamental rules that govern life's maintenance and adaptation might operate elsewhere. Understanding the origins and resilience of life is, after all, a physiological question at its deepest level.

In September 2025, when NASA announced evidence suggestive of past biological activity on Mars (NASA, 2025), the story made headlines around the world. It touched on two of the most profound scientific questions of all: Are we alone in the universe? and What is the origin of life? As The Physiological Society defines it, ‘Physiology is the science of life’, the branch of biology that seeks to understand the mechanisms underlying living systems. Yet, despite this focus, the concept of life itself remains elusive. More than 123 operational definitions are used in the literature, which share recurring themes of self‐organisation, metabolism, reproduction and evolution (Trifonov, 2011). This includes NASA's working definition, ‘a self‐sustaining chemical system capable of Darwinian evolution’, developed by its Exobiology Discipline Working Group in the mid‐1990s (Benner, 2010). However, no single, universally accepted consensus exists across scientific fields, beyond the tentative and broad notion of ‘self‐reproduction with variations’ (Trifonov, 2011).

‘It's a God‐awful small affair’, David Bowie (1947–2016) sang in 1971, yet the question posed in his song ‘Life on Mars?’ was anything but small; it strikes at one of the most profound problems in science. Indeed, any serious consideration of life on Mars must distinguish between extinct and extant life, because the planet's habitability has changed profoundly over geological time (Dzurilla & Teece, 2024). During the Noachian period (approximately 4.1–3.7 billion years ago), Mars possessed a thicker atmosphere, widespread surface water, valley networks, lakes and possibly a northern ocean, conditions broadly compatible with sustained surface habitability (Wordsworth, 2016). During the subsequent Hesperian (3.7–2.9 billion years ago), the global magnetosphere was gradually lost, which accelerated atmospheric escape, while surface temperatures fell, radiation exposure increased and volcanism drove sulfur‐rich, acidic environments, increasingly restricting liquid water to transient floods and subsurface reservoirs, and by the early Amazonian (from 2.9 billion years ago to the present), Mars had become predominantly cold and arid, with only episodic and localised evidence for liquid water, such as glacial melt or volcanically induced heating (Jakosky et al., 2018). The stable aqueous environments that might once have supported a surface biosphere largely disappeared, shifting any remaining habitability to subsurface niches. The most promising environments for such extant life on modern Mars are so‐called *special regions*, where sufficient solar or geothermal energy, temperature and pressure conditions may still permit liquid or solid water and levels of water activity compatible with microbial replication (Dzurilla & Teece, 2024).

The study that led to NASA's remarkable announcement pointed specifically towards extinct surface microbial life, based on analyses of samples collected by NASA's Perseverance rover (Figure 1) from the ancient lake bed of Jezero Crater dating back to the late Noachian to early Hesperian period. Mineral assemblages reveal that volcanic rocks were repeatedly altered by water, yielding oxidation–reduction (redox) gradients ideally suited to the kinds of chemical reactions that, on Earth, sustain microbial life (Hurowitz et al., 2025). These minerals occur together with organic carbon compounds in fine‐grained mudstones that also show redox fronts, implying ancient electron‐transfer activity at low temperatures. On Earth, such mineral–organic associations and redox interfaces are characteristic of habitats for chemolithotrophic microorganisms that draw energy from iron and sulfur compounds, and are therefore widely regarded as candidate biosignatures of past microbial metabolism.

**FIGURE 1:**
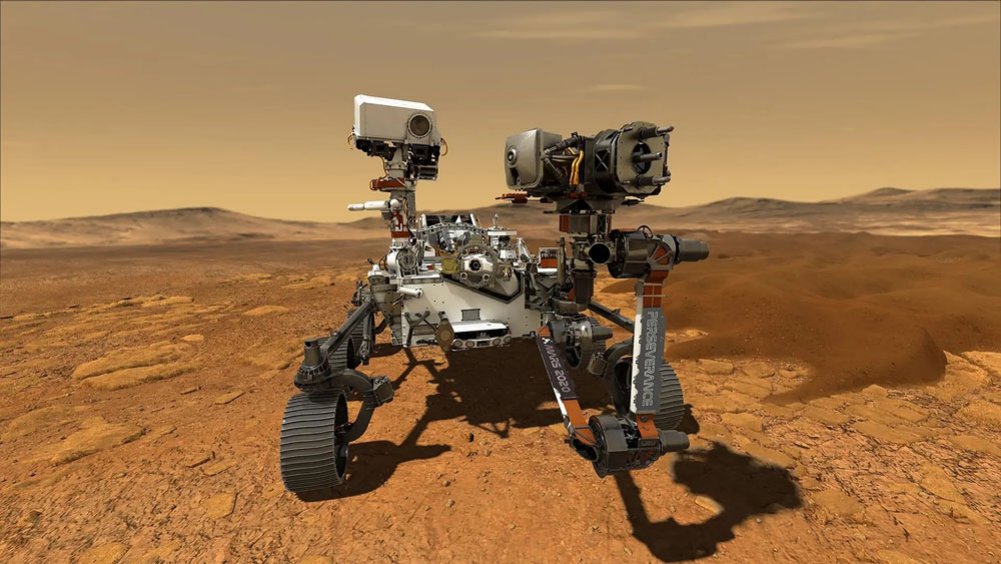
NASA's Perseverance rover. In Jezero Crater on Mars, Perseverance has collected samples from ancient lake‐bed deposits showing that Mars once hosted long‐lived aqueous systems. Mineral assemblages reveal that volcanic rocks were repeatedly altered by water, producing redox gradients ideally suited to the types of chemical reactions that, on Earth, sustain microbial life. Image credit: NASA/JPL‐Caltech.

Complementary evidence comes from NASA's Curiosity rover in Gale Crater, a 154‐km‐wide impact basin located approximately 3700 km south of Jezero Crater, where Perseverance operates. There, Curiosity drilled into a 3.7‐billion‐year‐old mudstone and detected the longest hydrocarbon chains yet found on Mars: decane, undecane and dodecane (Freissinet et al., 2025). These compounds are consistent with the thermal degradation of long‐chain fatty‐acid precursors, as might arise from the breakdown of biological lipids, although their ultimate origin, that is, abiotic or biological, remains unresolved. In any event, the redox gradients preserved in these rocks are, in essence, the same energetic differentials that underpin life's most fundamental processes today (Bailey, 2019). The electron‐transfer reactions that once operated at Martian water–rock interfaces closely resemble those that underpin mitochondrial respiration, vascular signalling and redox homeostasis in living tissues, mediated by electron carriers such as cytochromes and flavoproteins whose evolutionary origins can be traced back to ancient geochemical systems. In physiological terms, this speaks directly to homeostasis and allostasis: the capacity of living systems to maintain internal stability, and to do so not statically, but through adaptive regulatory adjustments in response to environmental challenge. Homeostasis reflects the preservation of key variables within viable limits, whereas allostasis captures the anticipatory and flexible recalibration of those limits under composite stress (Bailey et al., 2025). Together, they define a core hallmark of life. In this sense, the geochemistry that may have sparked the earliest metabolic processes continues to shape the molecular logic of modern physiology, governing how organisms sense, respond to and survive within changing environments.

While it may be disappointing to some that the evidence for Life on Mars does not point to ancient extinct organisms or advanced civilisations of the sort often portrayed in popular culture (Bailey, 2022), it does suggest the past presence of microbial life, perhaps in the form of biofilms or mats. These indications remind us that when the Solar System was formed approximately 4.6 billion years ago, all the fundamental building blocks of life were already present (Bailey, 2020). The formation of the Solar System began with the collapse of a presolar dust‐and‐gas cloud, likely triggered by the shockwave from a nearby Type II supernova (Podosek et al., 2000). The ejecta from that explosion seeded the cloud with heavier elements, including ^4^ ^0^K produced during oxygen burning in the dying star's core. Because ^4^ ^0^K has a half‐life of approximately 1.3 billion years, this radioactive trace of our supernovaic past remains present in every potassium‐containing compound on Earth and elsewhere in the Solar System to this day (Berg, 2023).

In terms of the molecular precursors of life, results from NASA's OSIRIS‐REx mission demonstrated that these are present on the surface of Bennu, a carbonaceous near‐Earth asteroid formed from the same primordial matter as the planets (Glavin et al., 2025). Samples from Bennu were returned to Earth in 2023, and analysis under ultra‐sterile laboratory conditions showed the presence of 33 amino acids, including 14 of the 20 used by life on Earth to build proteins, as well as all five nucleobases found in DNA and RNA.

However, a mandatory ingredient for life as we know it, whether on Earth, Mars or elsewhere, is water. According to the ‘giant‐impact’ astrogeological hypothesis (also known as the Theia Impact), a Mars‐sized protoplanet, often referred to as Theia, collided with the proto‐Earth approximately 4.47 billion years ago, ejecting material that later coalesced to form the Moon (Young et al., 2016). Isotopic analyses of hydrogen and other volatiles in both terrestrial and lunar materials indicate that Earth's water was largely retained from its original building blocks despite this cosmic collision, and to some extent accreted later from carbonaceous chondrites, that is primitive meteorites rich in hydrated minerals (Greenwood et al., 2018; Piani et al., 2020). Accordingly, isotopic evidence suggests that most of Earth's water predated the giant impact itself, and that a substantial portion of it resides deep within the planet. In 2014, the discovery of a microscopic inclusion of ringwoodite trapped within a diamond from Brazil revealed about 1% water by weight, implying that the mantle transition zone, lying between 410 and 660 km beneath the surface, may contain up to three times as much water as all of Earth's oceans combined (Pearson et al., 2014).

Subsequent seismic studies have demonstrated that regions near the top of the lower mantle exhibit patterns of partial melting consistent with the presence of water released from hydrated ringwoodite. These observations suggest that this mineral stores significant amounts of water deep within the Earth and that the mantle transition zone acts as a long‐term reservoir of bound water far exceeding that found at the surface (Schmandt et al., 2014). Unlike Earth, Mars lacked the plate tectonics and magnetic shielding needed to recycle and protect its early water inventory. The gradual loss of this internal reservoir to space may have ended any nascent biosphere before it could evolve complexity. While Mars lost its capacity to recycle water and sustain redox‐driven chemistry, Earth retained a dynamic hydrosphere that later became the stage for life's emergence.

Indeed, the water on Earth that formed the first oceans provided the base for a warm alkaline primordial soup containing carbon dioxide, hydrogen, ammonia, sulfides, phosphates and organic precursors such as amino acids, sugars, fatty acids and nucleotides, in which life began to take shape approximately 4 billion years ago. In the metal‐rich hydrothermal vents formed by serpentinising rocks on the ocean floor, this mixture was far from inert; it hosted a network of reactions that are today characteristic of life itself, including carbon fixation into small carboxylic acids, Krebs‐ and glycolysis‐like cycles and the synthesis of amino and nucleotide precursors, all proceeding spontaneously under the same temperature and redox conditions that still sustain vent microbes. These processes transformed simple geochemistry into protometabolism.

These networks laid the foundation for the first truly biological entities. The leap from protometabolism to biology is marked by the emergence of the Last Universal Common Ancestor (LUCA), not a single organism in the modern sense, but a population of simple, self‐replicating cells sharing a common biochemical architecture (Glansdorff et al., 2008). LUCA likely existed ∼3.8–4.0 billion years ago, thriving in hydrothermal environments rich in hydrogen, carbon dioxide and transition metals. Its molecular toolkit already included ribosomes, RNA‐based catalysis, ATP synthase and proton‐driven chemiosmosis, the same bioenergetic machinery that powers life today. Sequence analyses also suggest that LUCA was capable of detoxifying reactive oxygen species long before oxygen became abundant in the atmosphere or ocean, probably as a result of localised oxygen formation via abiotic sources (e.g. photolysis of water by ultraviolet light) or cohabitation with an oxidative photosynthesising organism (Bailey, 2019). From LUCA onward, life diversified through descent with modification, yet retained a unifying physiological grammar: redox chemistry, membrane gradients and information flow. Every mitochondrion, chloroplast and enzyme system can trace its ancestry to this shared origin. LUCA thus represents not only the root of the biological tree, but the first physiological system, that is, a self‐sustaining entity capable of maintaining internal gradients against an external flux.

The most profound transition, long viewed as the boundary between chemistry and biology is energy coupling: the formation of high‐energy phosphates such as ADP and ATP in aqueous environments without enzymatic machinery (De Duve, 1998). Because ATP functions as the universal energy currency of all known life, its emergence is often regarded as the foundational reaction of living systems. For decades it was assumed that such phosphorylation was impossible without enzymes, fuelling arguments that spontaneous life was implausible or required external intervention. However, new evidence, currently available as a preprint, suggests that reduced phosphorus species such as phosphite, generated in serpentinising hydrothermal systems, can phosphorylate AMP to ADP on native metal surfaces at alkaline water–rock interfaces (Mrnjavac et al., 2025). While this form of energy coupling is likely to have been essential, abiogenesis would also have required the parallel emergence of self‐replicating informational molecules capable of heredity and variation, most plausibly RNA‐like systems that predate DNA‐based biology and link early metabolism with molecular evolution (Joyce & Szostak, 2018).

Viewed through a physiological lens, these primitive systems can be regarded as the earliest expressions of adaptive homeostasis. Proton and electron gradients across mineral membranes acted as proto‐cellular boundaries, coupling energy flow to chemical order, with matter beginning to sense and respond to its own gradients; the earliest ‘whisper’ of physiology. What we now recognise as feedback and homeostasis were born as self‐organising chemical responses to flux. In this sense, metabolism preceded biology: physiology emerged first as physics and chemistry discovered how to regulate themselves. Today, synthetic biologists working to construct minimal cells from inorganic components are, in effect, replaying these same steps in reverse, demonstrating that physiology's origin story remains an unfinished experiment (Szostak et al., 2001).

The evolution of life can be regarded as a thermodynamically favourable phenomenon, a process of dissipation‐driven adaptation in which self‐organising systems emerge and evolve because they are efficient at capturing and dissipating energy from their surroundings (England, 2020). In contemporary physiology, these same principles of adaptation and energy efficiency, that is allostasis, are explored as we study how complex organisms (humans among them) cope with the cumulative stresses imposed by the space exposome which reflects the cumulative sum of all environmental hazards encountered during spaceflight (Bailey, 2026; Bailey & van Ombergen, 2026; Bailey et al., 2025): cosmic radiation, isolation and confinement, distance from Earth, hostile and closed environments, and altered gravity fields (Bailey, 2026). Just as early life evolved to harness and buffer its environment, modern physiology explores how life copes when those environmental constants are disrupted.

The recent findings discussed above suggest that life may emerge wherever appropriate substrates coincide with permissive environmental conditions on Earth, on Mars, or elsewhere among the countless stars above us. As Erwin Schrödinger (1887–1961) argued in his monograph *What is Life?*, living systems persist as flows of energy and information, maintaining matter far from equilibrium by feeding, as he memorably put it, on ‘negative entropy’ (Schrödinger, 1944). From this perspective, even when adhering to a minimal definition such as ‘self‐reproduction with variation’, the boundary between living and non‐living matter is less a sharp demarcation than a gradient: an evolutionary, and therefore probabilistic, continuum of increasing organisation, regulation and responsiveness. Life's emergence is thus unlikely to reflect a single canonical pathway, but rather a spectrum of likelihoods shaped by local physicochemical constraints over time. If the same forces that animated life on Earth also operated within Martian mudstones, then physiology is not merely the study of life on our planet; it is the study of how the universe comes to know itself – through us!

## AUTHOR CONTRIBUTIONS

Ronan Martin Griffin Berg and Damian Miles Bailey and wrote the first draft of the manuscript. Ronan Martin Griffin Berg and Damian Miles Bailey edited and revised the manuscript. Ronan Martin Griffin Berg and Damian Miles Bailey edited and revised the manuscript. Ronan Martin Griffin Berg and Damian Miles Bailey approved the final version submitted for publication and agree to be accountable for all aspects of the work in ensuring that questions related to the accuracy or integrity of any part of the work are appropriately investigated and resolved. All persons designated as authors qualify for authorship, and all those who qualify for authorship are listed.

## CONFLICT OF INTEREST

D.M.B. is Editor‐in‐Chief of *Experimental Physiology*, Chair of the Life Sciences Working Group, member of the Human Spaceflight and Exploration Science Advisory Committee to the European Space Agency, member of the Space Exploration Advisory Committee to the UK and Swedish National Space Agencies and member of the National Cardiovascular Network for Wales and South‐East Wales Vascular Network. R.M.G.B. is Deputy Editor‐in‐Chief (Europe) of *Experimental Physiology*.
